# Histone methylation modification patterns and relevant M-RiskScore in acute myeloid leukemia^☆^

**DOI:** 10.1016/j.heliyon.2022.e10610

**Published:** 2022-09-16

**Authors:** Dade Rong, Xiaomin Chen, Jing Xiao, Daiyuan Liu, Xiangna Ni, Xiuzhen Tong, Haihe Wang

**Affiliations:** a The First Affiliated Hospital, Sun Yat-sen University, 58 Second Zhongshan Road, Guangzhou, 510080, China; b Department of Biochemistry, Zhongshan School of Medicine, Sun Yat-sen University, 74 Second Zhongshan Road, Guangzhou, 510080, China; c Zhuhai Interventional Medical Center, Zhuhai Precision Medical Center, Department of Clinical Laboratory, Zhuhai People's Hospital (Zhuhai Hospital Affiliated with Jinan University), Zhuhai, 519000, China; d GenePlus, Beijing, China; e Faculty of Health Sciences, University of Macau, Macau, China

**Keywords:** Acute myeloid leukemia, Histone, Methylation, Classification, Chemotherapy

## Abstract

**Objective:**

We tried to identify novel molecular subtypes of acute myeloid leukemia (AML) associated with histone methylation and established a relevant scoring system to predict treatment response and prognosis of AML.

**Methods:**

Gene expression data and clinical characteristics of patients with AML were obtained from The Cancer Genome Atlas (TCGA) database and Gene Expression Omnibus (GEO) database. Molecular subtyping was carried out by consensus clustering analysis, based on the expression of 24 histone methylation modification regulators (HMMRs). The clinical and biological features of each clustered pattern were taken into account. The scoring system was constructed by using differential expression analysis, Cox regression method and lasso regression analysis. Subsequently, the scoring system in the roles of prognostic and chemotherapeutic prediction of AML were explored. Finally, an independent GSE dataset was used for validating the established clustering system.

**Results:**

Two distinct subtypes of AML were identified based on the expression of the 24 HMMRs, which exhibited remarkable differences in several clinical and biological characteristics, including HMMRs expression, AML-M0 distribution, *NPM1* mutation, tumor mutation burden, somatic mutations, pathway activation, immune cell infiltration and patient survival. The scoring system, M-RiskScore, was established. Integrated analysis demonstrated that patients with the low M-RiskScore displayed a prominent survival advantage and a good response to decitabine treatment, while patients with high M-RiskScore have resistance to decitabine, but they could benefit from IA regimen therapy.

**Conclusion:**

Detection of HMMRs expression would be a potential strategy for AML subtyping. Meanwhile, targeting histone methylation would be a preferred strategy for either AML-M0 or *NPM1* mutant patients. M-RiskScore was a useful prognostic biomarker and a guide for the choice of appropriate chemotherapy strategy.

## 1 Introduction

Acute myeloid leukemia (AML) is an age-distributed hematopoietic stem cell differentiation disorder with the inhibition of hematopoietic stem cell differentiation and the accumulation of immature cells at various stages, as well as the reduced production of active hematopoietic elements and cytokines (Ferrara and Schiffer, 2013; Short et al., 2018). Meanwhile, AML is also a highly heterogeneous disease attributed to various pathogenetic factors, including various chromosomal and molecular abnormalities (Papaemmanuil et al., 2016; Prada-Arismendy et al., 2017). Due to the progress in the development of new chemotherapeutic drugs, the application of allogeneic stem cell transplantation and the proposal and implementation of the supportive treatment, the overall prognosis of AML patients has been significantly improved (Ferrara and Schiffer, 2013). However, more than half of young adult patients and approximately 90% of elderly patients still die of the disease itself or the toxicity along with therapeutic drugs, and thereby the heterogeneity of this disease remains the main obstacle (Ferrara and Schiffer, 2013; Short et al., 2018).

Two main systems that have been used to classify AML into subtypes are the French-American-British (FAB) classification and the newer World Health Organization (WHO) classification (Hwang, 2020; Vardiman, 2012). FAB classification, a method that largely based on the morphological features of leukemia cells with routine staining and observation under a microscope, can comprehensively divided AML cases into subtypes M0-M7, based on the cell types, maturity or differentiation status of leukemia cells in blood or bone marrow (Hwang, 2020; Vardiman, 2012). However, this FAB classification contributes a limited role in guiding AML patient therapy, as so far, there are almost no differences in the treatment regimens for other subtypes, except AML-M3 subtype (de Thé et al., 2017; Hwang, 2020; Vardiman, 2012). The World Health Organization (WHO) classification of AML was established in 2001 with the advances in sequencing technology (Jaffe et al., 2001). After two updates in 2008 and 2016, respectively, the WHO system divides AML into 11 subtypes, based on chromosomal and genetic mutations in AML cells (Arber et al., 2016; Vardiman et al., 2009). In comparison with the FAB classification, the WHO system displays more advantages in guiding individual treatment, because it takes account many of the factors that affect the diagnosis and prognosis of AML (Hwang, 2020; Vardiman, 2012). Nevertheless, the WHO classification does not cluster AML comprehensively, due to the heterogeneity of AML cells during leukemogenesis.

Epigenetics refers to the covalent modification of DNA, RNA and histones without changing the DNA sequence to affect the expression of genes (Egger et al., 2004). Epigenetic inhibitor therapy usually targets DNA methylation (decitabine and azacytidine) and histone acetylation (Chidamide) to benefit AML patients. Epigenetic inhibitors not only prove the value of targeting epigenetic regulators for AML treatment but also urges understanding of epigenetic regulation and the discovery of novel targets for effective AML treatment (Ball et al., 2017; Cashen et al., 2010; Stahl et al., 2018; Tsai and So, 2017; Wang et al., 2020). Histone methylation modification is one type of epigenetic modification that alters chromatin structure by methylating the lysine or arginine residues in histone tails to activate gene expression or silence it (Audia and Campbell, 2016; Cheung and So, 2011; Hammond et al., 2017). Similar to other epigenetic modifications, there are three types of regulators involved in histone methylation modification, namely histone methyltransferases (“Writers”), histone demethylases (“Erasers”) and histone methyl modification recognition factors (“Readers”), respectively (Audia and Campbell, 2016). In recent years, emerging studies have shown that aberrant histone methylation is closely related to leukemogenesis (Salvatori et al., 2011; Schenk et al., 2012). Meanwhile, a series of lead compounds targeting histone methylation regulators have been completed in the preclinical and even entered clinical studies (Kruger et al., 2013; Maes et al., 2018; Swords et al., 2015; Tsai and So, 2017; Wouters and Delwel, 2016). However, the current studies on the use of histone methylation modification in AML are limited, attributed to the fewer changes in one or a few single genes, while the mutation patterns in genes related to leukemia often involve multiple genes that interact in a highly coordinated manner in clinical practice (Ferrara and Schiffer, 2013; Short et al., 2018). Therefore, it is necessary to systematically study the expression and characteristics of histone methylation regulators in AML, which would favor the diagnosis and treatment of AML.

In this study, we established a comprehensive classification approach for AML based on the expression level of 24 HMMRs in AML patients. At the same time, we identified two distinct histone methylation modification patterns that exhibit remarkable differences in several clinical and biological characteristics, including AML-M0 distribution, mutations of *NPM1*, survival, TMB, somatic mutations, and pathways activation and immune cell infiltration. Besides, based on the clustering, we established a novel scoring system, M-RiskScore, which not only acts as an independent prognostic predictor but also guides an appropriate and effective chemotherapy strategy.

## 2 Materials and methods

### 2.1 Data collection

Gene expression, mutation and clinical annotation data of AML cases were obtained in The Cancer Genome Atlas (TCGA) database (https://portal.gdc.cancer.gov/) and Gene Expression Omnibus (GEO) database (https://www.ncbi.nlm.nih.gov/geo/). LAML cohort from the TCGA database was used as the training dataset. GSE110087, GSE84334, GSE103424 and GSE71014 cohorts were acquired from the GEO database. Among them, the first three cohorts were used for analyses of chemotherapy in AML, and the last one was for validation.

24 HMMRs, including 13 writers (KMT2A, KMT2D, KMT5A, SETD2, NSD1, SMYD3, NSD2, DOT1L, EZH2, SETD7, CARM1, SUV39H1, EHMT2), 7 erasers (KDM1A, KDM2A, KDM4A, KDM5A, KDM5B, KDM6A, KDM6B) and 4 readers (ATRX, EED, PC, RAG2), were chosen and identified from the related studies for further analyses (Audia and Campbell, 2016).

### 2.2 Landscape for histone methylation modification regulators in AML

Correlation analysis of the gene expression among the 24 histone methylation modification regulators in AML was explored by the “corrplot” package. The Wilcoxon rank-sum test was used to investigate the expression difference of histone methylome regulators in AML patients with different RiskStatus. Information on somatic mutation and copy number variations (CNV) of included genes were generated from cBioPortal website (https://www.cbioportal.org/).

### 2.3 Consensus clustering of 24 histone methylation modification regulators

We conducted the Consensus clustering analysis to characterize and identify distinct histone methylation modification patterns in AML cases based on the expression of the above-mentioned 24 histone methylome regulators to classify AML patients into possible subtypes for further analysis. The number of clusters and their stability was determined by the consensus clustering algorithm, and 1000 times repetitions were conducted for guaranteeing the stability of classification (Wilkerson and Hayes, 2010).

### 2.4 Features of distinct histone methylation modification patterns

A series of analyses were performed to validate the histone methylation modification patterns after finishing the consensus clustering. Principal component analysis (PCA), a technique for reducing the dimensionality of such datasets, increasing interpretability but at the same time minimizing information loss (Jolliffe and Cadima, 2016), was conducted to verify the quality of consensus clustering. The “Survival” package was applied to explore the time-dependent prognostic value of the clusters.

Tumor mutational burden (TMB), a new promising biomarker that emerged recently, is classically defined as the number of non-synonymous exonic mutations per megabase (Mb) (Fumet et al., 2020). The total number of mutations counted was divided by the exome size (38 Mb was utilized as the exome size), by which we calculated the TMB of each case. TMB correlation analysis was executed to explore the association between TMB and the distinct clusters.

To discriminate the biological activity difference between the clustered AML subtypes, we performed gene set variation analysis (GSVA) enrichment analysis by using “GSVA” R packages, which is an unsupervised and non-parametric method for estimating the variation in biological process and pathway activity in the samples of an expression dataset (Hänzelmann et al., 2013). The gene sets of “h.all.v7.1.symbols” were downloaded from the MSigDB database (https://www.gsea-msigdb.org/gsea/index.jsp) for GSVA analysis. The result was considered to be statistically significant while its p-value was less than 0.05. Moreover, we explored the somatic gene mutations in the different clusters by the “maftools” package.

### 2.5 Estimation of immune cell infiltration

We explored immune cell infiltration patterns between the distinct AML clusters with MCP-counter and CIBERSORT methods. MCP-counter is a methodology based on transcriptomic markers that assess the proportion of immune and stromal cell populations in the tumor microenvironment (TME) from transcriptomic data. There are 10 cell populations estimated by MCP-counter, including T cells, CD8+ T cells, cytotoxic lymphocytes, B lineage, NK cells, Myeloid dendritic cells, Neutrophils, Endothelial cells and Fibroblasts (Becht et al., 2016). CIBERSORT, a known deconvolution algorithm, is used to quantify the 22 infiltrated immune cells, according to the normalized gene expression profiles. The 22 immune cells are composed of memory B cells, naive B cells, plasma cells, resting/activated DCs, resting/activated NK cells, resting/activated mast cells, eosinophils, neutrophils, monocytes, M0–M2 macrophages, and 7 T-cell types (CD8+ T cells, regulatory T cells (Tregs), resting/activated memory CD4+ T cells, follicular helper T cells, naive CD4+ T cells and γδ T cells) (Newman et al., 2015).

Another 20 immune checkpoint genes (*CD244, PDCD1, PD-L1, CTLA4, CD80, CD86, CD28, TIGIT, PVR, CD96, SIRPA, CD47, LGALS9, HAVCR2, ICOS, ICOSLG, TNFSF18, TNFRSF18, KLRD1, KLRC1*) were retrieved from a previous study (Burugu et al., 2018), and correlation analysis was conducted to determine the differential expression of immune checkpoint genes between the two characterized AML clusters.

### 2.6 Construction of histone methylation modification-related score (M-RiskScore) in AML

We first conducted the empirical Bayesian approach with the limma package to determine the differentially expressed genes (DEGs) among the histone methylation modification patterns of AML cases. Then, we performed a univariate Cox regression analysis to identify the prognostic genes with a p-value less than 0.01 for further analysis.

The lasso regression analysis was subsequently applied to construct the histone methylation modification-related score (RiskScore) by “glmnet” and “survival” packages. In this analysis, a lasso penalty was used to account for shrinkage and variable selection. The optimal value of the lambda penalty parameter was defined by performing 10 cross-validations. The calculation formula for the acetylation-related score was as follows:

RiskScore=(coefficientmRNA1×expressionofmRNA1)

+(coefficientmRNA2×expressionofmRNA2)+⋅⋅⋅

+(coefficientmRNAn×expressionofmRNAn)

According to the median of the RiskScore, AML patients were divided into two groups (high-risk or low-risk group). We then performed the survival analysis based on this grouping strategy. A receiver operating characteristic (ROC) curve, which is a plot of the sensitivity versus 1 − specificity of a diagnostic test (Mandrekar, 2010), was constructed to examine the prognostic accuracy to verify the RiskScore. Finally, to validate whether the RiskScore could be an independent prognostic marker in AML, we carried out the univariate and multivariate Cox regression analyses.

### 2.7 Nomogram construction and validation

To improve the clinical application of RiskScore, we constructed the nomogram based on the Cox regression model. There were 5 components of the nomogram, including gender, FAB subtype, RiskStatus, age, and RiskScore. Decision curve analysis was performed to compare the net benefits of different prognostic models (FAB subtype, RiskStatus, RiskScore and nomogram). The concordance index, calibration plot, and ROC curve were used to verify the nomogram.

Nomograms are a pictorial representation of a complex mathematical formula (Grimes, 2008). Medical nomograms use biological and clinical variables, such as tumor grade and patient age, to determine a statistical prognostic model that generates a probability of a clinical event, such as cancer recurrence or death, for a particular individual (Balachandran et al., 2015).

Model performance was evaluated through calibration and discrimination (Alba et al., 2017). Bias corrected calibration for 3 years and 5 years overall survival rate was performed by 1000 bootstrap resamples to evaluate the consistency between the observed and estimated survival probability with “rms” package. Discrimination was evaluated by Harrell's concordance index (C-index) and ROC curve. A higher C-index value demonstrated better model-fitting performance. Area under the ROC curve (AUC) value is an effective way to summarize the overall diagnostic accuracy of the test, taking values from 0 to 1, where a value of 0 indicates a perfectly inaccurate test and a value of 1 reflects a perfectly accurate test, indicating a higher AUC value revealed superior model discriminative ability (Mandrekar, 2010).

Decision curve analysis (DCA) was further performed to measure and compare the clinical utilities of the different prognostic models. DCA is a method for evaluating the benefit of a diagnosis test across a range of patient preferences for accepting the risk of undertreatment and overtreatment to facilitate decisions about test selection and use (Fitzgerald et al., 2015).

### 2.8 Biological characteristics of RiskScore

GSVA analysis, TMB analysis, Somatic gene mutations analysis and immune cell infiltration analysis were performed to exhibit the biological characteristics of the high-RiskScore group and low-RiskScore group, respectively.

## 3 Results

### 3.1 Landscape of genetic variation of histone methylation modification regulators in AML

**Figure 1:**
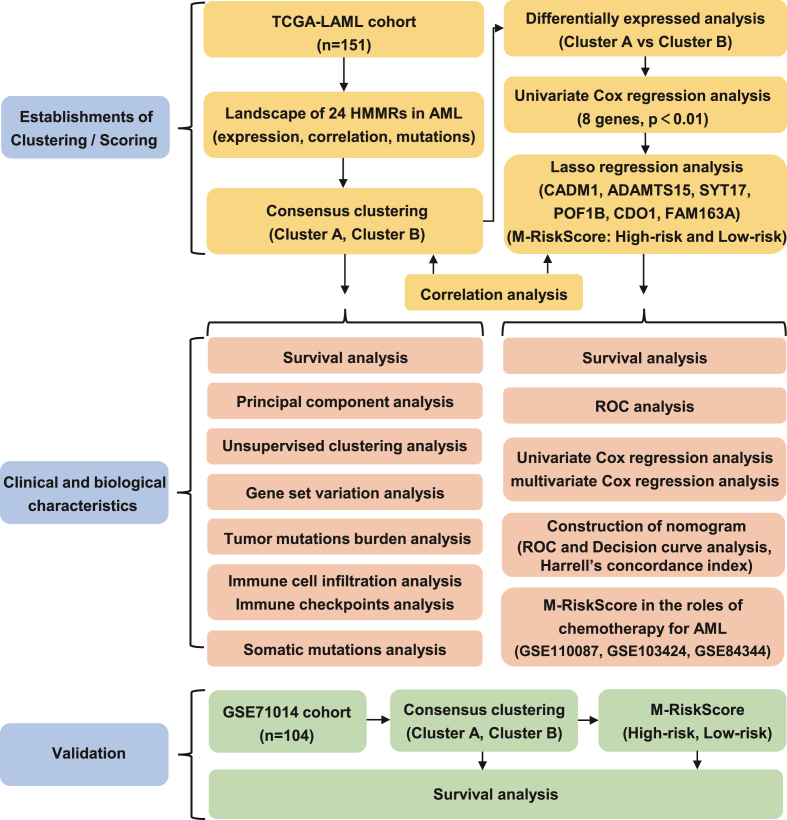
Study flowchart.

The overview of this work is shown in the form of a flowchart (Figure 1). The clinical profiling of TCGA-LAML cohorts was summarized in Table 1.

**Table 1:** Clinical characteristics of AML cohort for classification and validation.

Clinical Characteristics		Number	Percent (%)
TCGA-LAML (n = 124)			
Survival status	Survival	47	37.9
	Death	77	62.1
Age	≥60 years	51	41.1
	<60 years	73	58.9
Gender	Female	57	46.0
	Male	67	54.0
IDH2-R132 mutation	Negative	112	90.3
	Positive	12	9.7
IDH2-R140 mutation	Negative	114	91.9
	Positive	10	8.1
IDH2-R172 mutation	Negative	122	98.4
	Positive	2	1.6
NPM1 mutation	Negative	93	75.0
	Positive	31	25.0
FLT3 mutation	Negative	88	71.0
	Positive	36	29.0
Activating RAS mutation	Negative	116	93.5
	Positive	8	6.5
FAB subtype	M0	12	9.7
	M1	30	24.2
	M2	27	21.8
	M3	13	10.4
	M4	27	21.8
	M5	12	9.7
	M6	2	1.6
	M7	1	0.8
Risk status	Favorable	28	22.6
	Intermediate	71	57.3
	Poor	25	20.1
GEO-GSE71014 (n = 104)			
Survival status	Survival	68	65.4
	Death	36	34.6

**Figure 2:**
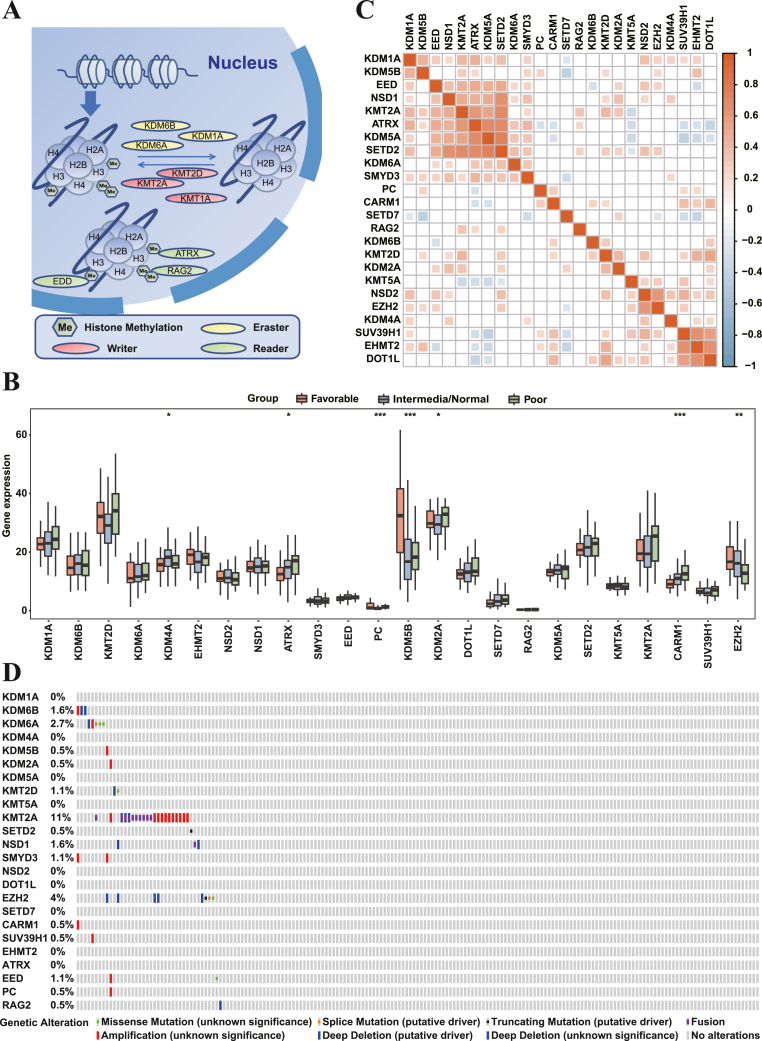
Landscape of genetic variation of 24 HMMRs in AML. A. Graphical summary of the dynamic process of histone methylation modification. B. The expression of 24 HMMRs among AML with different Risk statuses. ∗p < 0.05; ∗∗p < 0.01; ∗∗∗p < 0.001. C. Co-expression analysis of 24 HMMRs in AML. Positive co-expression, red; Negative co-expression, blue. D. Overview of somatic mutations and chromosomal variations of 24 HMMRs in AML.

A total of 24 HMMRs were finally identified in this study, including 13 writers, 7 erasers and 4 readers. The dynamic reversible process of histone methylation mediated by HMMRs was summarized in Figure 2A. We analyzed the gene expression profile of these 24 regulators in AML patients regarding their RiskStatus level, and results showed that there were 6 regulators endorsed with remarkable gene expression uniqueness in RiskStatus, including 1 writer (CARM1), 2 readers (ATRX and PC) and 3 erasers (KDM2A, KDM4A and KDM5B) (Figure 2B). Among them, up-regulation of ATRX, PC, KDM2A and CARM1 featured a poor RiskStatus in AML progression and prognosis, while up-regulation of EZH2 and KDM5B in turn suggested a favorable RiskStatus feature (Figure 2B).

Given the interplaying among HMMRs, we performed a co-expression analysis and demonstrated that there were two positive co-expression zones (red boxes), in which the bigger one displayed that SETD2, a histone methyltransferase, has a positive correlation with ATRX, KDM5A, NSD1 and KMT2A in gene expression, respectively (Figure 2C). Meanwhile, the 3 writers (SUV39H1, DOT1L and EHMT2) that contained in the smaller box form a co-expression loop (Figure 2C). A significant negative correlation in gene expression was unveiled between KDM5B and SETD7, KDM5A and SUV39H1, along with ATRX and DOT1L, respectively (Figure 2C). Eventually, we summarized the incidence of the somatic mutation and copy number variation (CNV) of 24 regulators in AML patients and showed no obvious relationship among them in terms of genetic alteration in other regulators, except KMT2A that exhibited a significant CNV alteration, including fusion and amplification in copy number (Figure 2D).

### 3.2 Histone methylation modification patterns mediated by 24 regulators in AML

**Figure 3:**
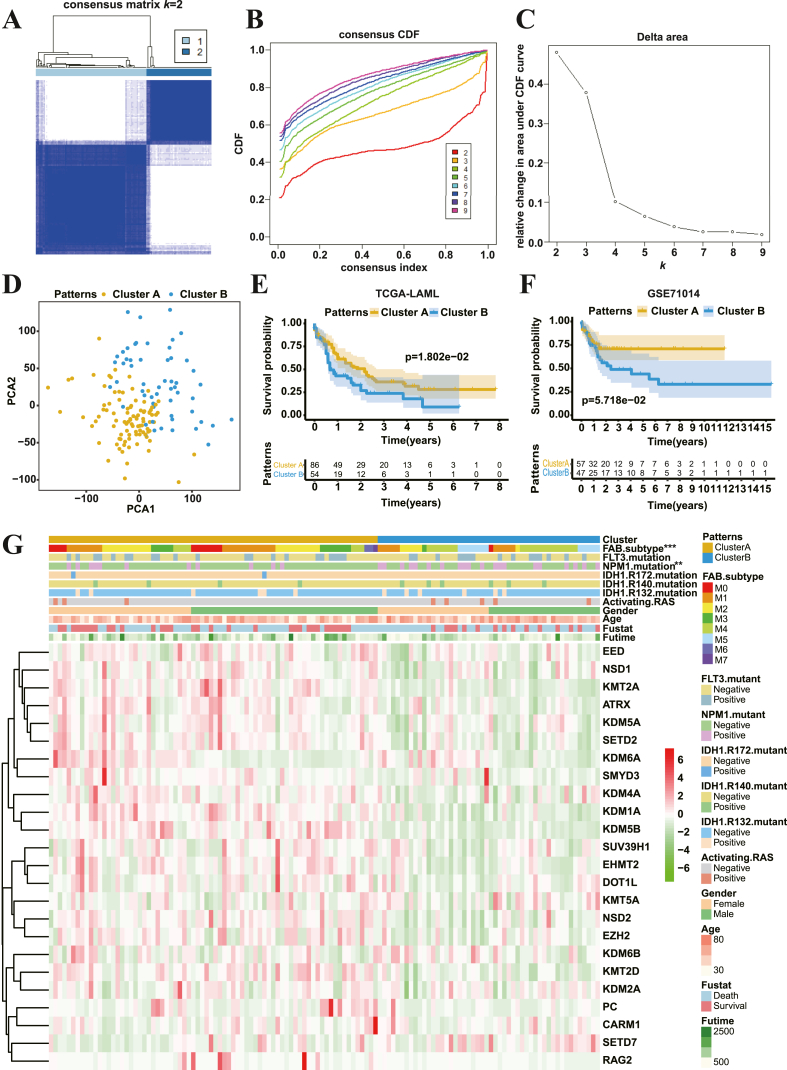
The establishment of histone methylation modification patterns in AML. A. Consensus matrix of TCGA-LAML cohort for k value is equal to 2. B. Cumulative distribution function (CDF) plot of the consensus matrices for *k* = 2–9. C. Delta area plot of CDF plot. D. Principal component analysis for the transcriptome profiles of the two histone methylation modification patterns. E. Survival analysis for the two histone methylation modification patterns of TCGA-LAML cohort. F. Survival analysis for the two histone methylation modification patterns of GSE71014 cohort. G. Unsupervised clustering of 24 HMMRs in the TCGA-LAML cohort. The histone methylation modification patterns, FAB subtype, activating RAS, gender, age, fustant, fustime and mutations of genes (FLT3, NPM1, IDH1-R172, IDH1-R140, IDH1-R132) were used as annotation. ∗∗p < 0.01; ∗∗∗p < 0.001.

To verify whether these 24 HMMRs can be used to classify the AML cases, the R package of ConsensusClusterPlus was approached to classify the patients with qualitatively measured histone methylation modification patterns based on the expression levels of these 24 HMMRs. Results showed that the optimal consensus clustering can be obtained when the *K* value of the consensus matrix was set as 2 (Figure 3A, S1). This result was further confirmed by the result of the Consensus Cumulative Distribution Function (CDF) Plot (Figure 3B) and Delta Area Plot (Figure 3C). Taken together, two clearly distinct histone methylation modification patterns of AML subtypes were eventually identified, 95 cases in patterns A and 56 cases in patterns B. We assigned these two patterns as Cluster A and Cluster B, respectively. Principal component analysis (PCA) of the transcriptome profiles of these two modification patterns was also performed and showed that there was a significant distinction existed between them (Figure 3D). To validate these established histone methylation modification AML patterns, we repeated the correlation analyses by using another independent AML-cohort, GSE71014, whose clinical profiles were summarized in Table 1. As shown in Figure S2A-C, the best consensus clustering was also obtained when the *K* value of the consensus matrix was set as 2, which was consistent with the above-mentioned clustering analyses (Figure 3A-D, S1). Moreover, the survival analysis displayed that the patients of Cluster A had a more prominent survival advantage than those of Cluster B in both TCGA-LAML and GSE71014 cohorts (Figure 3E-F).

Subsequently, unsupervised clustering of 24 HMMRs was conducted to explore the clinical features of these two histone methylation modification AML patterns in TCGA-LAML cohort annotated with factors/features of FAB subtypes, typical gene mutations, gender, age, fustat and futime. As shown in Figure 3G and S3, the gene expression levels of most of the 24 HHMRs were remarkably upregulated in Cluster A, compared with Cluster B. Thus, Cluster A showed an up-regulation tendency in these 24 genes overall (Figure 3G and S3). It is worth noting that there was a significant discrepancy between Cluster A and Cluster B in terms of the distribution of FAB category, in which the AML-M0 subtype was only distributed in Cluster A, but not in Cluster B. Besides, more patients with *NPM1* mutation distributed in Cluster B than Cluster A. However, there were no obvious differences between the patients in Cluster A and Cluster B with features of *FLT3* mutation, *IDH1* mutation, activating *Ras*, gender, age, fustat and futime.

### 3.3 Biological characteristics of distinct histone methylation modification patterns in AML

**Figure 4:**
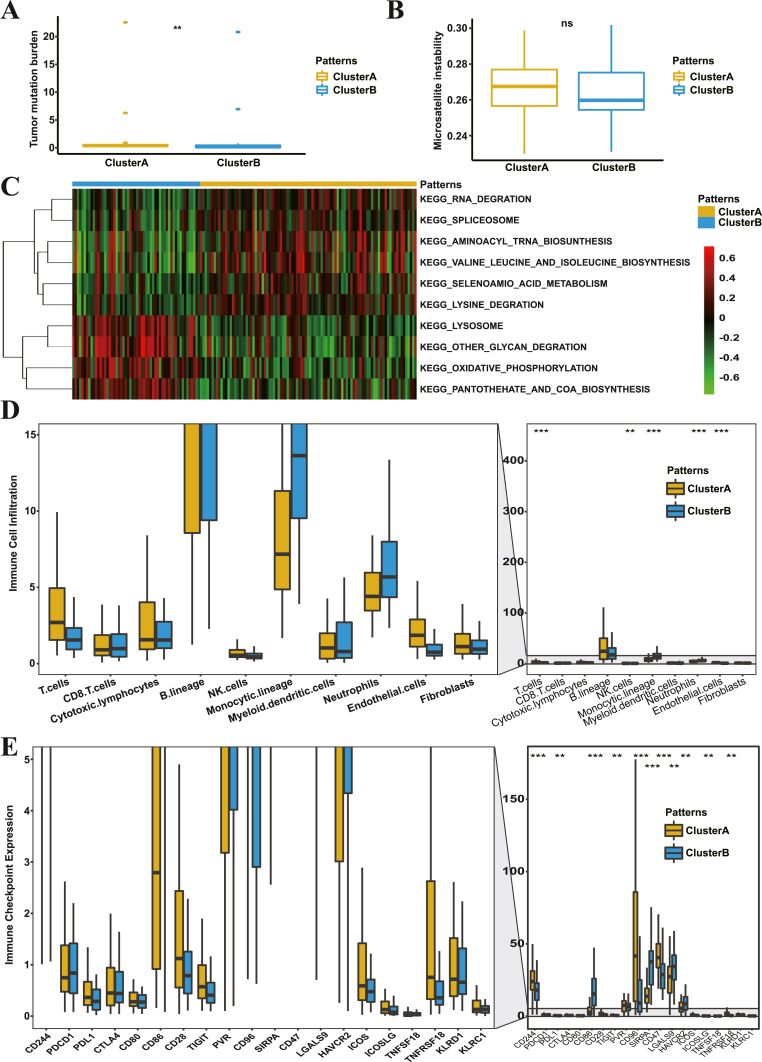
Biological characteristics of the two AML histone methylation modification patterns. A. Tumor mutation burden analysis of two histone methylation modification AML patterns. ∗∗p < 0.01. B. Microsatellite instability analysis for the above two histone methylation modification patterns. ns: no statistically significant. C. GSVA enrichment analysis shows the activation states of biological pathways in the distinct histone methylation modification patterns. The heatmap was used to visualize these biological processes. Activated pathways were represented in red and inhibited pathways were represented in blue. D. The abundance of each TME infiltrating cell in the two histone methylation modification patterns. ∗∗p < 0.01; ∗∗∗p < 0.001. E. The expression of typical immune checkpoint proteins between the two histone methylation modification patterns. ∗∗p < 0.01; ∗∗∗p < 0.001.

To determine the biological character distinction between Cluster A and Cluster B, we first analyzed their distribution of somatic mutations in TCGA-AML cohort with maftools package. The result of somatic mutation analysis showed that Cluster A presented a more extensive tumor mutation burden (TMB) than Cluster B, with the altering frequency 62.5% against 48.15% (Figure S4A-B). Furthermore, the tumor mutation burden quantification analysis also confirmed that Cluster A was markedly correlated with a higher TMB level (Figure 4A), although no significant difference in microsatellite instability was observed between Cluster A and Cluster B (Figure 4B). To further investigate the physiological characteristics of Cluster A and Cluster B, we performed GSVA enrichment analysis and manifested that Cluster A showed a remarkable enrichment in protein and RNA metabolism pathways, including RNA degradation pathway, spliceosome pathway, valine leucine and isoleucine biosynthesis pathways, and so on (Figure 4C). Whereas, Cluster B presented enrichment pathways associated with energy metabolisms, such as oxidative phosphorylation, pantothenate and CoA biosynthesis and lysosome pathways (Figure 4C).

The tumor microenvironment (TME), composed of tumor cells, stromal cells, immune cells and multiple secreted factors, plays a crucial role in tumor progression (Hinshaw and Shevde, 2019; Yuan, 2016). We then performed the MCP-counter method to examine the immune cell infiltration status of these two AML clusters. Results revealed that Cluster A cases presented a higher immune cell infiltration of T cells, B cells, NK cells and endothelial cells, but a lower infiltration of monocytes and neutrophils that were the noumenon of tumor cells (Figure 4D). Then, we conducted the CIBERSORT method to verify the result of the MCP-counter method. Consistently, the result also showed that the infiltration of CD4+ memory T cells, naive B cells, resting NK cells and plasma cells in Cluster A were all higher than those in Cluster B, except for the lower infiltration of monocytes (Figure S4C).

Enlightened by the results of immune cell infiltration, we compared the expression of some immune cell markers, chemokines and cytokines between Cluster A and Cluster B to figure out more about the immune characteristics of histone methylation modification patterns in AML. As shown in Figure 4E, a series of immune-activating factors were up-regulated in Cluster A, including CD244 which is a marker of NK cells, CD96 which plays a role in the adhesive interaction of activated T and NK cells, inducible T cell costimulatory ligand (ISOSLG), TNFRSF18 and CD47, in comparison with Cluster B. At the same time, some immune-inhibiting factors were downregulated in Cluster A, including HAVCR2, CD86 and LGALS9. Taken the previous results that the expression of most of the 24 HMMRs were generally upregulated in Cluster A (Figure 3G), we speculated HMMRs might play a role in the active expression of a series of immune-activating factors in AML.

### 3.4 Establishment of histone methylation modification-related M-RiskScore in AML

**Figure 5:**
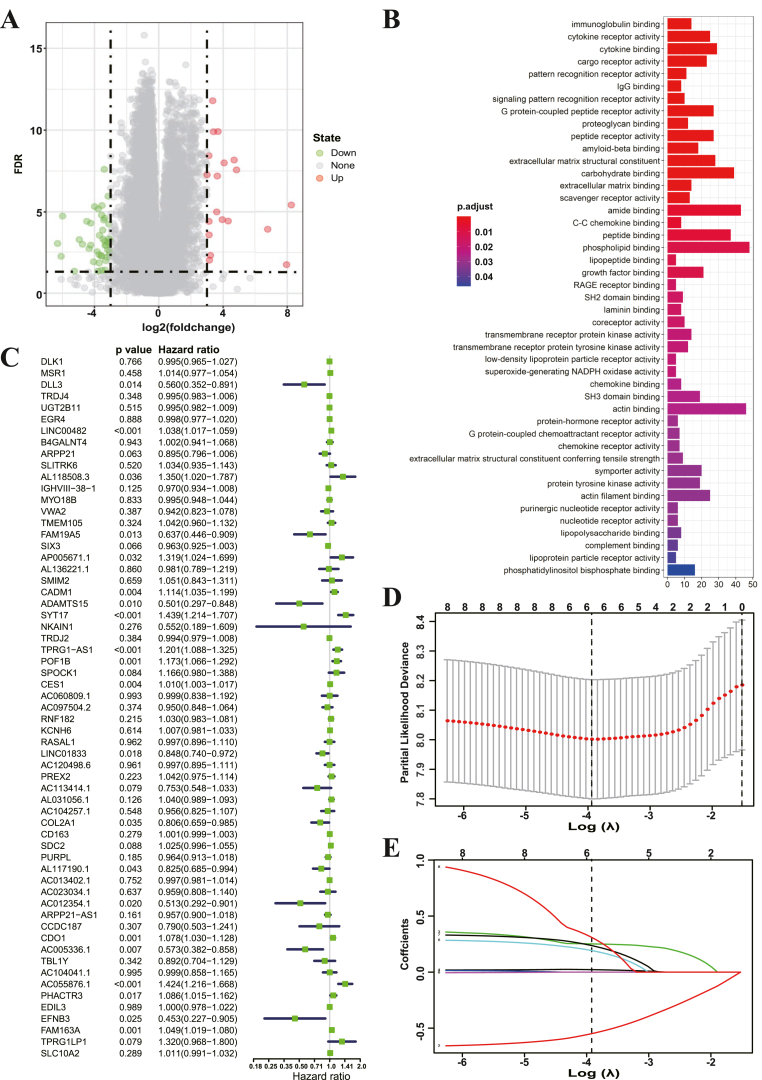
Establishment of M-RiskScore system. A. A volcano plot showing the differential expression genes (DEGs) between the two histone methylation modification patterns. The value of log2 fold change was set as ±3. B. Functional annotation of 61 DEGs with GO enrichment analysis. The color depth of the bar plots represents the activation of the pathways. The length of the bar plots represents the number of the genes enriched. C. The prognostic analyses of 61 DEGs in the TCGA-LAML cohort with a univariate Cox regression model. The genes highlighted were selected for lasso regression analysis. D and E. Results of lasso regression analysis. The determination of the value of λ (D) and the determination of the coefficient values of the selected genes from C (E).

To further evaluate the changes in transcriptome of Cluster A and Cluster B, we conducted the empirical Bayesian approach of the limma package to separate the differentially expressed genes (DEGs) between these two clusters. The volcano plots exhibited that there were lots of DEGs between Cluster A and Cluster B, in which 61 DEGs even with the log2 (fold change) value up to ±3 (Figure 5A). The clusterProfiler package was also used to perform GO enrichment analysis for these DEGs. As shown in Figure 5B, the immune system-related pathways were directly enriched in Cluster A, rather than Cluster B, indicating again that histone methylation modification plays a non-negligible role in regulating TME landscapes in AML.

To directly predict the individual therapeutic effect and prognosis of the patients with AML basing on the histone methylation modification patterns, we established a scoring system to quantify the histone methylation modification alteration of AML patients based on the indicated DEGs in Figure 5A. We termed this scoring system as M-RiskScore. Univariate Cox analysis identified the genes that are eligible for lasso regression analysis and 8 genes were further selected as marker genes, all of which possessed meaningful p-value (less than 0.01) (Figure 5C). The lasso regression analysis drew a formula for calculating the M-RishScore, in which 6 genes were finally included, including ADAMTS15, CADM1, CDO1, SYT17, FAM163A and POF1B (Figure 5D-E).

### 3.5 M-RiskScore is effective in prognosis prediction of AML

**Figure 6:**
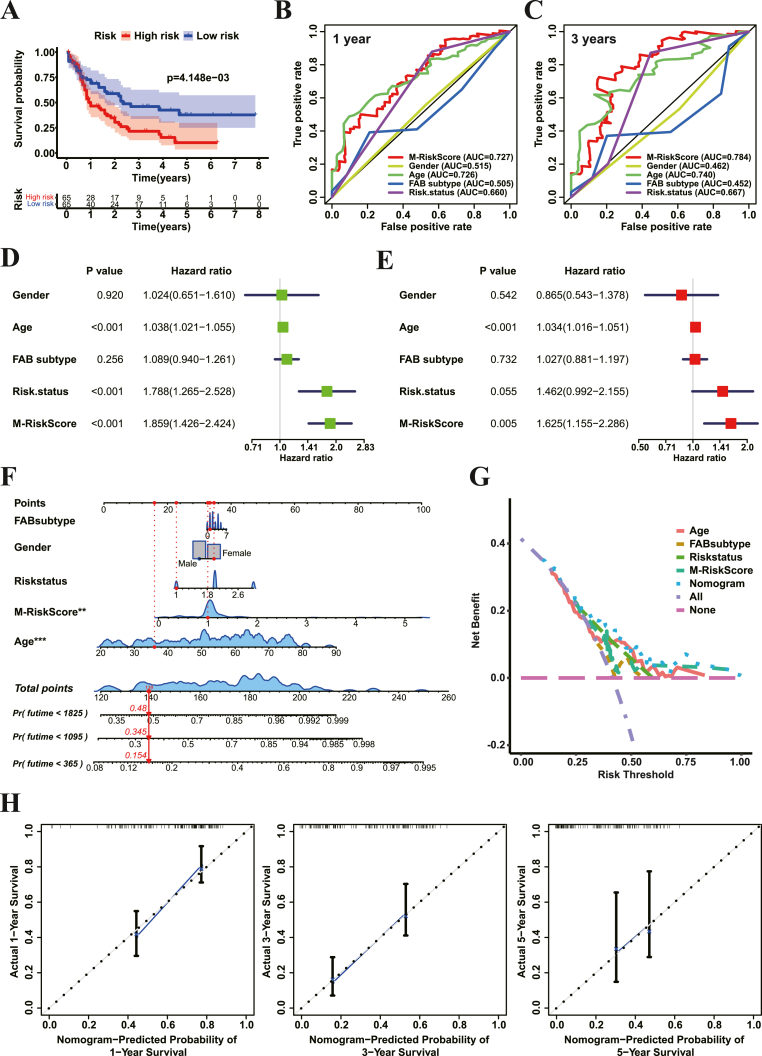
Application of M-RiskScore in the prognostic prediction of AML A. Survival analysis of the low and high M-RiskScore patient groups with TCGA-LAML cohort. B–C. ROC analyses of prognostic prediction accuracy of 3-year survival rate (B) and 5-year survival rate (C) of M-RiskScore, Gender, Age, FAB subtype and Riskstatus in TCGA-LAML cohort. D-E. Univariate Cox regression model (D) and multivariate Cox regression model (E) for prognostic analyses of M-RiskScore, Gender, Age, FAB subtype and Riskstatus in TCGA-LAML cohort. F. Construction of nomogram composed of M-RiskScore, Gender, Age, FAB subtype and Risk status. G. The decision curve analysis of nomogram and its components. H. The calibration plot of a nomogram of the probability of survival of 1 year, 3 years and 5 years, respectively.

To explore the potential practical value of our M-RiskScore system in predicting the outcome of AML patients, we divided all AML patients into the high and low M-RiskScore groups when the medium value was set as the cutoff value. Prognosis analysis showed that the low M-RiskScore group exhibited a better survival rate than the high M-RiskScore group (Figure 6A). Impressively, the results of correlation analysis between histone methylation modification patterns and M-RiskScore displayed that Cluster A possessed a lower M-RiskScore (Figure S5A), indicating the reliable and practical importance of M-RiskScore.

To validate the prediction accuracy of M-RiskScore, two receiver-operating characteristics (ROC) curves were created. As shown in Figure 6B-C, M-RiskScore exhibited the highest AUC (area under the curve) value in both the cohorts of 3-years and 5-years, verifying its remarkable predictive accuracy compared with other prognostic markers of AML. In addition, results of univariate and multivariate Cox regression analyses showed that the Hazard ratio of M-RiskScore was significantly higher than those with other prognostic markers of AML (Figure 6D-E), suggesting the independent and accurate potential role of M-RiskScore system in AML patient prognosis.

To further improve the prognostic application of M-RiskScore in AML, we performed the nomogram associating M-RiskScore with other prognostic markers of AML, including FAB subtype, gender, RiskStatus and age (Figure 6F). Results of the decision curve analysis (DCA) from the nomogram indicated that M-RiskScore had a robotic higher Net Benefit value than those independent prognostic markers in terms of the Risk Threshold range (Figure 6G). Moreover, all the calibration plots for the probability of survival of 1-year, 3-year and 5-year ran very close to the diagonal with an outstanding calibration effect (Figure 6H). However, the result of the ROC analysis of nomogram exhibited no notable increase in the prediction accuracy, compared with independent M-RiskScore in both the cohorts of 3-years and 5-years (Figure 6B-C, Figure S5B).

### 3.6 M-RiskScore is useful in the chemotherapy regimen decision of AML patients

**Figure 7:**
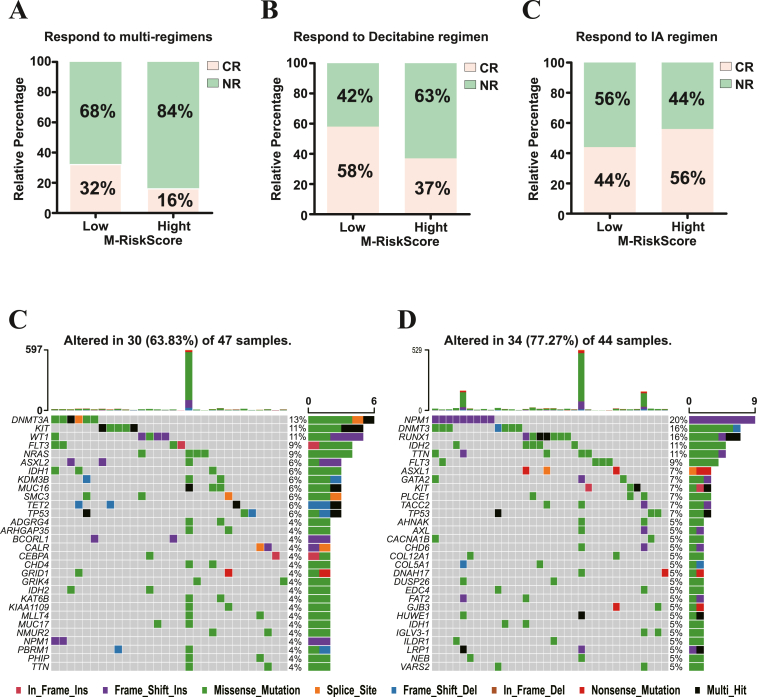
Validation of M-RiskScore in chemotherapy of AML. A. Correlation analysis of M-RiskScore with complex-chemotherapeutic response in GSE110087-AML cohort. B. Correlation analysis of M-RiskScore with decitabine treatment response in GSE84334-AML cohort. C. Correlation analysis of M-RiskScore with IA regimen treatment response in GSE103424-AML cohort. D. Somatic mutation analysis of low (left) and high (right) M-RiskScore patients' groups with AML progression in TCGA-LAML cohort.

Chemotherapeutic drugs still play an important roles in AML treatment due to their universal and highly effective cytotoxicity (Short et al., 2018). Thus, we tested the potential of M-RiskScore in predicting the efficacy of chemotherapy in AML patients. Three GEO-AML cohorts accompanying with chemotherapeutic information were downloaded and analyzed here. They were GSE84344 with the response information of decitabine treatment, GSE103424 with clinical characteristics before and after IA regimen (idarubicin + cytarabine) treatment, and GSE10087 with clinical information after different standard induction chemotherapy, including IA regimen, CIA regimen (clofarabine + idarubicin + cytarabine), FAI regimen (fludarabine + cytarabine + idarubicin), decitabine, BID FA regimen (twice daily fludarabine + cytarabine) and CECA regimen (cyclophosphamide + etoposide + carboplatin + cytarabine). The basic clinical characteristics of these three GEO-AML cohorts were summarized in Table 2. Results revealed that patients with low M-RiskScore possessed more obvious therapeutic advantages in both the complex-chemotherapy-treatment and the decitabine-treatment, compared to those with high M-RiskScore (Figure 7A-B). In contrast, AML patients with high M-RiskScore benefited more from the IA regimen treatment (Figure 7C). The somatic mutation analysis of AML-TCGA cohort displayed that the three most frequently mutated genes of the patients with low M-RiskScore were *DNMT3A*, *KIT* and *WT1*. In contrast, in the high M-RiskScore group, *NPM1*, *DNMT3A* and *RUNX1* were observed the most frequently mutated genes (Figure 7D-E). DNMT3A, a DNA methyltransferase encoded by *DNMT3A*, is one of the well-known targets of decitabine (Watts and Nimer, 2018), we speculated that the high mutation frequency of DNMT3A would be the authentic cause of poor chemotherapeutic response to decitabine treatment in the high M-RiskScore group (Figure 7B, D). Likely, we suspected that the mutated *KIT* and/or mutated *WT1* could play a crucial role in the progression of low M-RiskScore AML patients, while mutations of *NPM1* and/or *RUNX1* endows worse progression in high M-RiskScore patients.

**Table 2:** Clinical characteristics of AML cohort for correlation analysis.

Patient series	GSE103424	GSE103424	GSE103424
**No. of patients**	52	45	41
**Age**			
≥60 years	NA	45	25
<60 years	NA	0	16
**Gender**			
Female	24	17	14
Male	28	28	27
**Risk status**			
Favorable	5	0	4
Intermediate	30	23	15
Poor	10	14	22
NA	7	8	0
**Response to chemotherapy**			
Yes	17	18	11
No	35	27	30

## 4 Discussions

Individualized treatment is the ultimate and ideal goal of cancer treatment, which can effectively alleviate the disease, minimize the risk of complications or side effects and is economical, basing on a comprehensive consideration of various physiological or clinical characteristics of the patients, including gender, age, genetic characteristics, and treatment histories (Buettner et al., 2013; Mendelsohn, 2013; Senft et al., 2017). The achievement of individualized cancer therapy requires both extensive pathologic subtyping of the tumors for their heterogeneity and diagnosis of genome alterations of the cancers (Senft et al., 2017). Advances in sequencing technique provide unprecedented views of the complex genetic and nongenetic heterogeneity within individual tumors, which not only discloses the alterations of gene expression of the patients, but also provide an opportunity for tumor subtyping or classification (Buettner et al., 2013; Mendelsohn, 2013; Senft et al., 2017).

With the abundant sequence information of AML cases of various public cohorts, we established a repeatable clustering and scoring system of AML based on 24 HMMRs' expression levels of the patients with AML. Patients with low expression of 24 HMMRs were mainly divided into a cluster, exhibiting a better survival advantage, a higher TMB value and a more significant immune cell infiltration, compared with those who possessed a higher expression of related genes in another independent cluster. Among them, cell adhesion molecule 1 (CADM1) is an apoptosis-inducing tumor suppressor that is inactivated by methylation in a variety of tumor types, which may play a role in chemotherapy-induced cell death in AML (Fisser et al., 2015), but the other 5 genes’ role was rarely reported in AML. ADAMTS15 is used to predict the survival of human breast carcinoma with the inhibitory functions to tumor growth and invasion of colorectal cancers (Porter et al., 2006; Viloria et al., 2009). The high FAM163A expression is associated with short survival time in hepatocellular carcinoma and affects the occurrence and development of neuroblastoma (Chen et al., 2022; Qiao et al., 2019). The methylation status of CDO1 was specifically high in small bowel cancer (SBC) (Kojima et al., 2019), which indicates it is a biomarker of SBC. It is also overexpressed in Sezary syndrome (SS), a rare, aggressive CD4+ cutaneous T-cell lymphoma (Booken et al., 2008). SYT17 is down-expressed in the responders vaccinated with tumor-loaded dendritic cells (DCs) than in the non-responders of indolent non-Hodgkin lymphoma (iNHL) (Fucà et al., 2021). POF1B is a cytoplasmic actin-binding protein involved in the regulation of cell adhesion, but no information on AML (Crespi et al., 2015; Lacombe et al., 2006). Thus, it is likely that these genes may also play important roles in AML, although the underlying mechanism is yet to be uncovered. A close connection between the histone methylation modification profile and M-RiskScore is validated with the obvious overlap of the patient cluster and the M-RiskScore classification. The remarkable survival advantage of patients is observed in the lowe M-RiskScore group, indicating the impressive role of M-RiskScore as a dependent prognostic marker in AML. Moreover, M-RiskScore also can serve as a diagnostic index for chemotherapy strategy.

FAB classification is the first and the most comprehensive classification method of AML in morphology (Vardiman, 2012). However, only AML-M3, which also referred to acute promyelocytic leukemia (APL), can be specifically cured with the combination treatment of retinoic acid (RA) with arsenic (de Thé et al., 2017), the other seven subtypes still share similar treatment regimens. Interestingly, we showed that almost all patients of AML-M0 subtype based on FAB classification were divided into Cluster A. As AML-M0 indicates acute leukemia with minimal sign of myeloid differentiation (Vardiman, 2012), we deduced that the activity of HMMR expression profile could be closely associated with AML-M0 occurrence. Consistent with our findings, compounds specific to key histone methylation-modifying enzymes were developed in AML management (Tsai and So, 2017), indicating that targeting histone methylome would have the potential for AML-M0 patients.

The mutation status of *NPM1* is well-known to be associated with leukemogenesis and is an important marker for the WHO classification of AML. We find here that patients with the high M-RiskScore have frequently *NPM1* mutation (as the top three mutated genes), hinting that *NPM1* mutation would be associated with the inactive gene expression of HMMRs in AML. Coincidently, it is reported that SP2509, a KDM1A antagonist, induces more apoptosis in mutant *NPM1*-expressing AML cells than those with mixed-lineage leukemia fusion oncoproteins (Fiskus et al., 2014). Similarly, the inhibitor of histone methyltransferase DOT1L exhibits potent cytotoxicity to the *NPM1*-mutated AML cells (Zhang et al., 2018). But more study is still needed to be conducted to ascertain the causality between mutation of *NPM1* and the inactive expression of HMMRs. We believe that these HMMRs are promising targets for *NPM1*-mutated AML therapy.

Likely, we speculate that the mutation of *RUNX1 (RUN family transcription factor 1)*, which also is a distinct factor of the WHO classification of AML (Bullinger et al., 2017) and one of the top three mutated genes of the patients with high M-RiskScore, contributes to the leukemogenesis of patients with high M-RiskScore individually or in combination with mutant *NPM1*. In contrast, mutated *KIT (KIT proto-oncogene, receptor tyrosine kinase)* and/or *WT1 (WT1 transcription factor)* would be the oncogenic cause of AML patients with low M-RiskScore, thus they should be included as the potent entities for AML classification, as studies have shown that mutated *KIT* and *WT1* are critically associated with leukemogenesis (Ayatollahi et al., 2017; Coombs et al., 2016; Jawhar et al., 2019; Niktoreh et al., 2019; Pronier et al., 2018; Rocquain et al., 2010; Wang et al., 2015).

Given that RUNX1 and WT1 are both transcription factors for a broad spectrum of genes, directly disrupting their function causes unbearable side effects. However, it is possible to conduct drug development of their target gene-encoding proteins, especially enzymes. KIT, a protein tyrosine kinase receptor, would be a good and direct chemotherapy target for AMLpatients. Imatinib, an inhibitor of BCR-ABL tyrosine kinase inhibitors (TKI) and the first-line chemotherapeutic drug for most patients with chronic myelogenous leukemia (CML) (Braun et al., 2020; Moslehi and Deininger, 2015), exhibits an effective inhibition on KIT kinase activity (Guida et al., 2007; Heinrich et al., 2000), implying its potential therapeutic application to the *KIT*-mutated AML patients.

Our results further demonstrate that patients with low M-RiskScore have a better response to the chemotherapy in an AML-cohort, implying that the lower the M-RiskScore is, the better chemotherapeutic response would outcome, especially to the treatment of decitabine, rather than IA therapy regimen. As the cohorts used in our study here do not contain abundant patient samples and differences between different races, there is no statistical difference in the current analysis. Nevertheless, our results still indicate that M-RiskScore is a novel and accurate prognostic or diagnostic marker for AML patients.

## 5 Conclusion

In conclusion, our study demonstrates that histone methylation modification profile plays a curial role in the diagnosis and prognosis of AML, and our M-RiskScore system could be a valuable tool not only for the classification of AML but also for the therapy strategy of the patients with AML.

## Declarations

### Author contribution statement

Dade Rong and Xiaomin Chen: Conceived and designed the experiments; Performed the experiments; Analyzed and interpreted the data; Wrote the paper.

Jing Xiao: Conceived and designed the experiments; Analyzed and interpreted the data; contributed reagents, materials, analysis tools or data; Wrote the paper.

Daiyuan Liu and Xiangna Ni: Conceived and designed the experiments; Analyzed and interpreted the data; Wrote the paper.

Xiuzhen Tong and Haihe Wang: Conceived and designed the experiments; contributed reagents, materials, analysis tools or data; Wrote the paper.

### Funding statement

Xiuzhen Tong was supported by Guangdong Basic and Applied Basic Research Foundation [No. 2019A1515010294].

Jing Xiao was supported by Guangdong Provincial Key Laboratory of Tumor Interventional Diagnosis and Treatment [2021B1212040004].

Haihe Wang was supported by National Natural Science Foundation of China [No. 81472730].

### Data availability statement

Data will be made available on request.

### Declaration of interests statement

The authors declare no conflict of interest.

### Additional information

No additional information is available for this paper.

## Acknowledgements

We are grateful to Wen-Chien Chou (Department of Laboratory Medicine, National Taiwan University Hospital, Taiwan), Luciano Castiello (Pasteur Italy Institute, Italy), Chieh-Lin Jerry Teng (Division of Hematology/Medical Oncology, Taichung Veterans General Hospital, Taiwan) and Stephan Rainer Bohl (Internal Medicine III, University Hospital of Ulm, Germany) for shared the genetic and clinical information of the patients with AML.

## Ethics approval and consent to participate

Not applicable.
