# Supplementary material for: Histone methylation modification patterns and relevant M-RiskScore in acute myeloid leukemia
Source: Heliyon. 2022 Sep 16;8(9):e10610. doi: 10.1016/j.heliyon.2022.e10610 (PMC9508520; doi:10.1016/j.heliyon.2022.e10610)
Supplement: Supplementary [file mmc1.pdf]

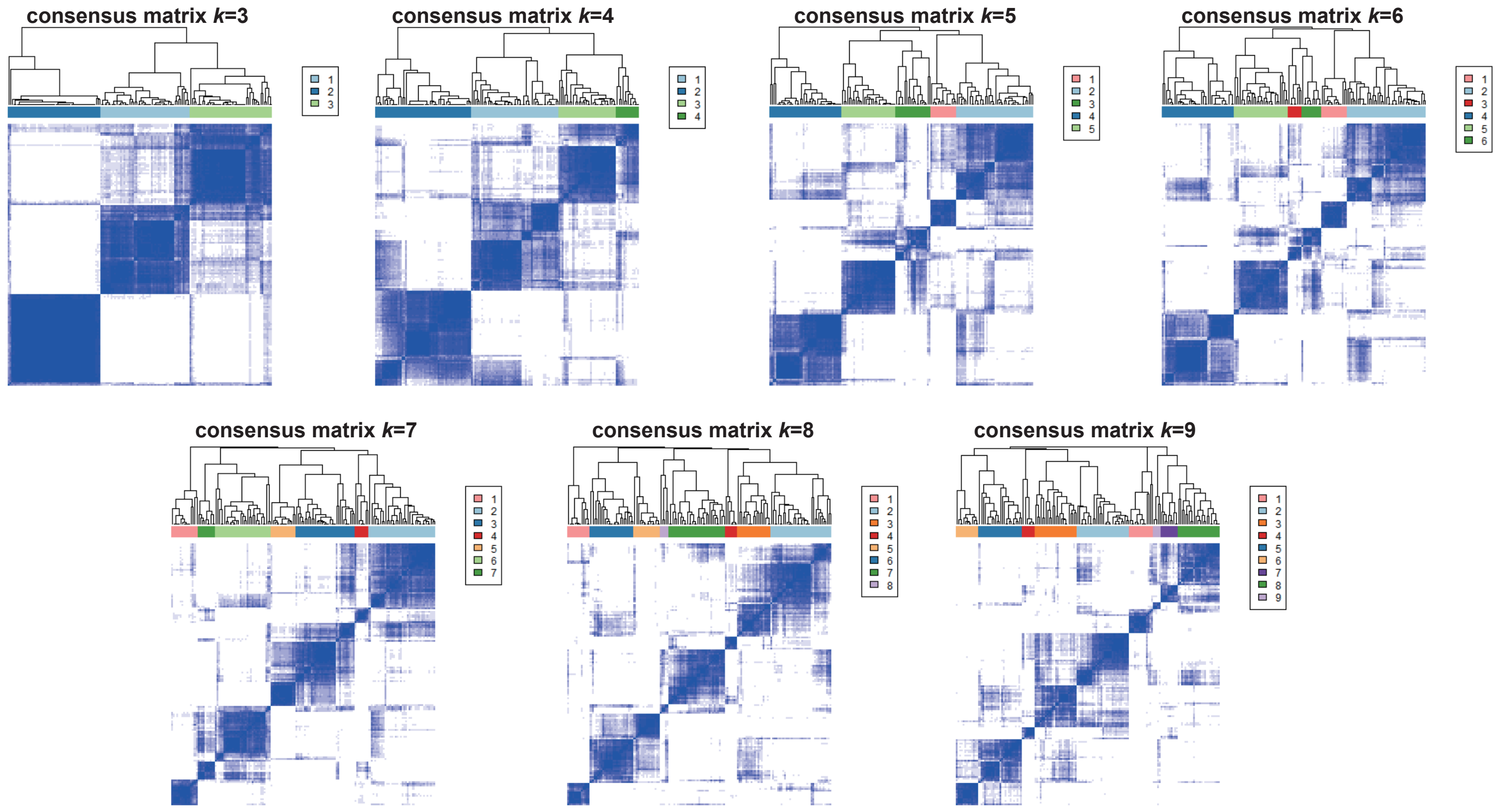

Figure S1. Consensus matrices of TCGA-LAML cohort for *k* value is equal to 3-9, respectively.

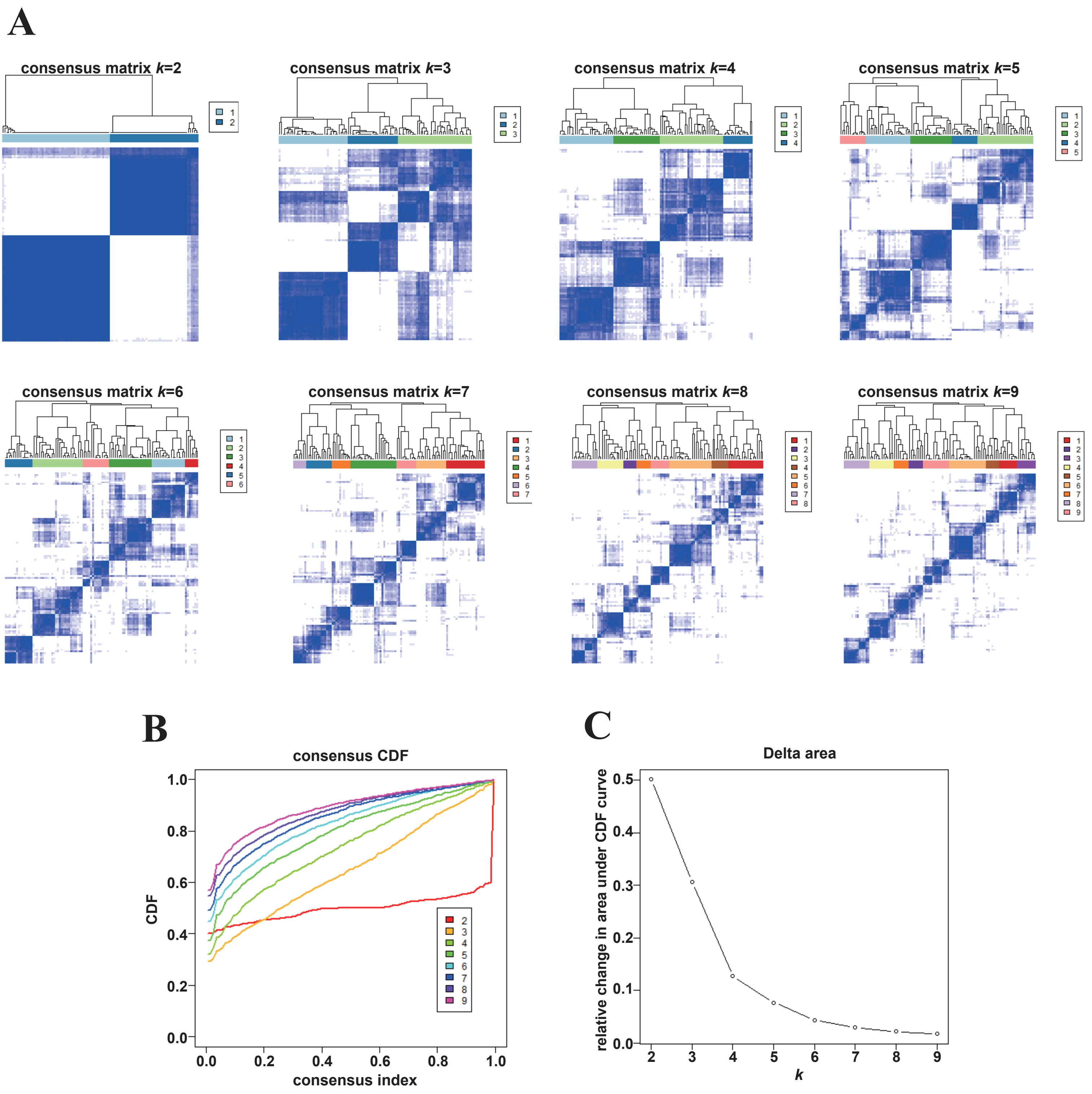

Figure S2. Validation of clustering.
(A) Consensus matrices of GSE71014 cohort for k = 2-9.
(B) Cumulative distribution function (CDF) plot of the consensus matrices for k = 2-9.
(C) Delta area plot of CDF plot.

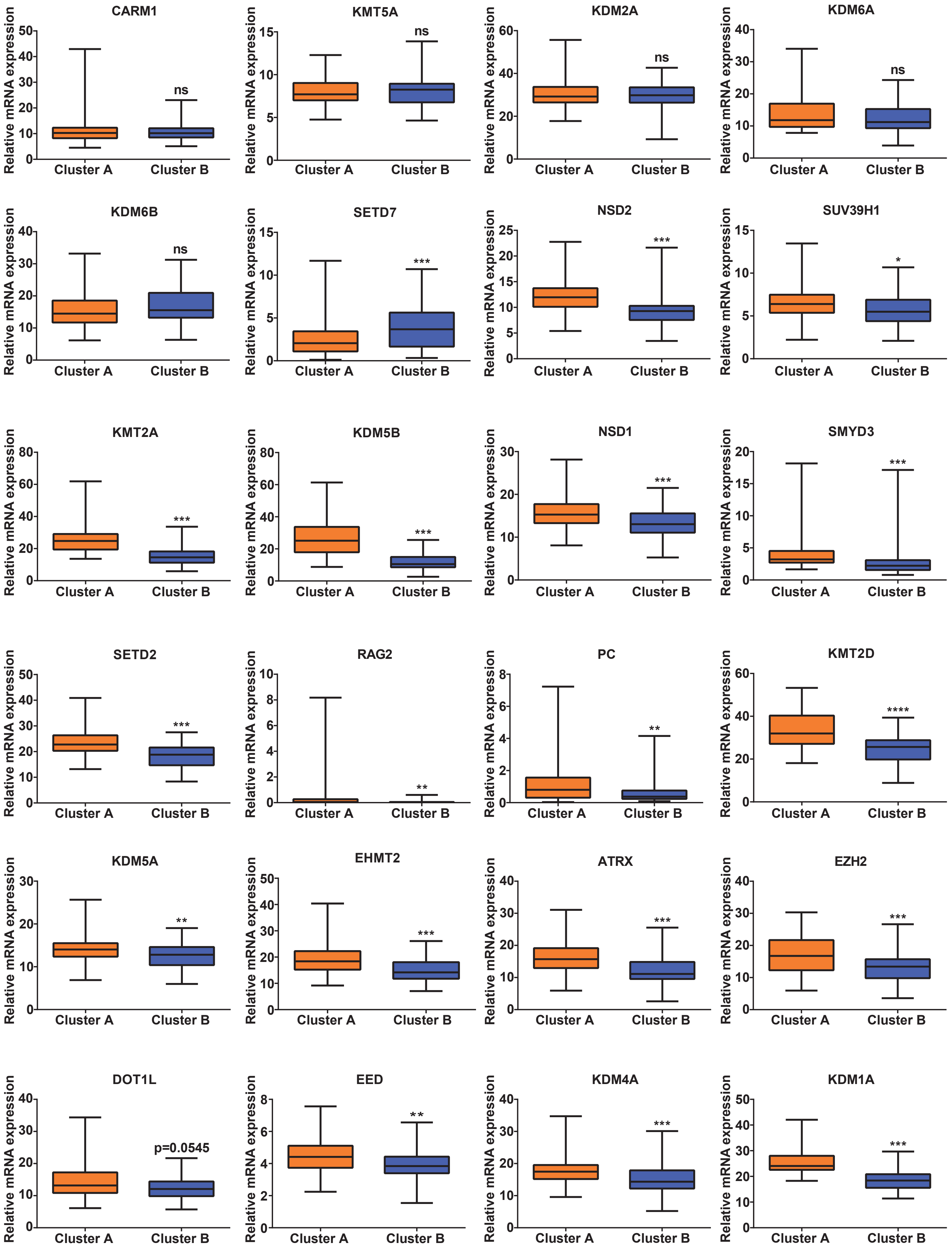

Figure S3. Relative mRNA expressions of 21 histone methylation modification regulators in Cluster A and Cluster B.

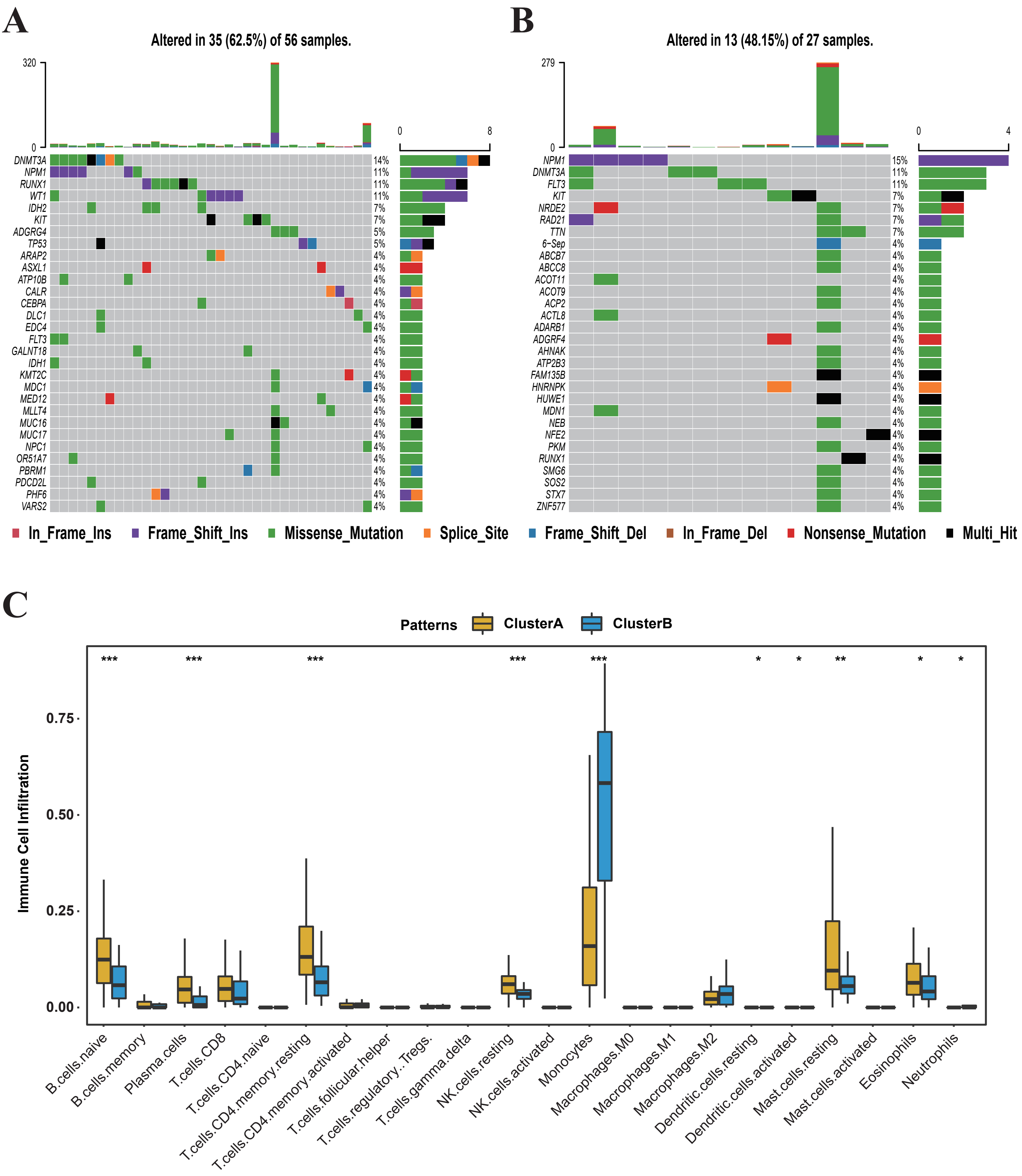

Figure S4. Biological characteristics of two distinct histone methylation modification patterns.
(A-B) Somatic mutation analyses for Cluster A (A) and Cluster B (B).
(C) Immune cells infiltration of two histone methylation modification patterns. * p<0.05; ** p<0.01; *** p<0.001.

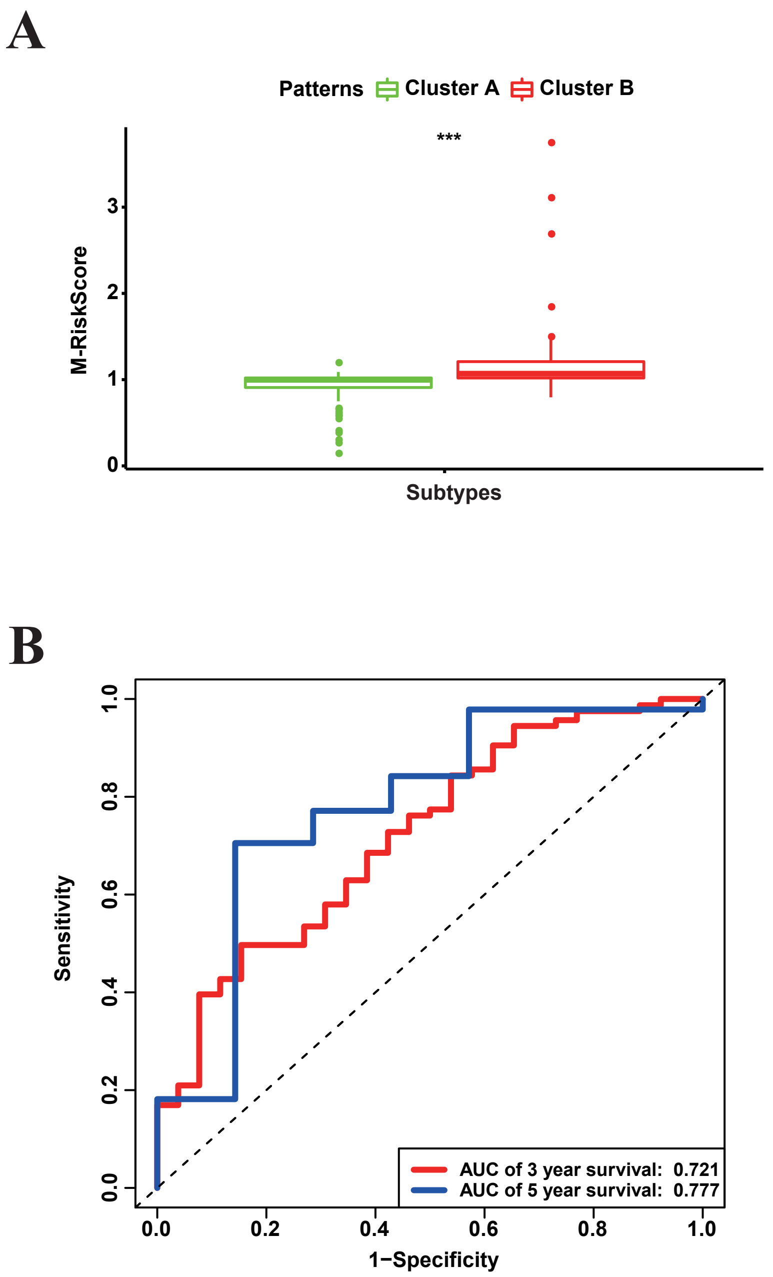

Figure S5. Correlation analysis of M-RiskScore and the two distinct patterns and ROC analysis of nomogram.

(A) Correlation analysis of M-RiskScore and the two histone methylation modification patterns.

(B) ROC analysis of nomogram.
